# Temporal Variations in Chemical Composition, In Vitro Digestibility, and Metabolizable Energy of Plant Species Browsed by Goats in Southern Mediterranean Forest Rangeland

**DOI:** 10.3390/ani11051441

**Published:** 2021-05-18

**Authors:** Youssef Chebli, Samira El Otmani, Mouad Chentouf, Jean-Luc Hornick, Jean-François Cabaraux

**Affiliations:** 1 Department of Veterinary Management of Animal Resources, University of Liège, Avenue de Cureghem 6, B43, 4000 Liège, Belgium; selotmani@doct.uliege.be (S.E.O.); jlhornick@uliege.be (J.-L.H.); jfcabaraux@uliege.be (J.-F.C.); 2 National Institute of Agricultural Research (INRA), 78 Bd. Mohamed Ben Abdellah, Tangier 90010, Morocco; mouad.chentouf@inra.ma

**Keywords:** forest rangeland, nutritive value, chemical composition, digestibility, goat, Southern Mediterranean

## Abstract

**Simple Summary:**

Mediterranean forest rangelands constitute essential feed resources for grazing goats. The objective of this study was to evaluate the temporal variations in chemical composition, in vitro digestibility, and metabolizable energy of browsed plant species by goats on forest rangelands of the Southern Mediterranean of northern Morocco. Overall, the nutritive value of the selected plant species was highest in spring and then steadily decreased through the summer and autumn. Most of the selected plant species present high levels of crude protein than the minimum required level for maintenance. This study provides a valuable and useful database to elaborate the seasonal grazing and feeding management plan for goat herds.

**Abstract:**

Forest rangelands contribute largely to goat diets in the Mediterranean area. Information about browsed plant quality is essential for adequate feeding management. The purpose of this study was to evaluate the temporal changes in chemical composition and in vitro digestibility of the main plant species selected by goats in the Southern Mediterranean forest rangeland during two consecutive years; these were very contrasted (dry and wet). The browsed species were composed of herbaceous, eleven shrubs, and four tree species. Overall, large variability in chemical composition, in vitro organic matter digestibility (IVOMD), and metabolizable energy (ME) was observed among species, grazing season (spring, summer, and autumn), and years within each species. Crude protein (CP) content varied from 60 to 240 g/kg dry matter (DM). The fiber fractions, except for *Quercus suber*, increased significantly by advancing maturity. Due to the water stress, the lignin level presented a higher value during the spring of the dry year. Condensed tannin (CT) content varied from 2 to 184 g/kg DM. CP, IVOMD, and ME showed a negative correlation with lignin and CT. Based on the results presented herein, it is concluded that the nutritive value of the browsed plant species was highest in the spring and lowest during the summer and autumn of both studied years. With a good grazing management strategy, the selected plant species by goats could guarantee high-quality feeding resources throughout the year.

## 1. Introduction

Mediterranean forests are composite landscapes of shrubs and trees, which constitute essential dietary resources for domestic ruminants. They also play a very important role in sustaining biodiversity [1] and provide multiple ecosystem services to local people for millennia [2]. These woodlands are characterized by heterogeneous and diversified flora [3].

Livestock, especially extensive goat farming, is one of the most important components of agricultural systems in the Southern Mediterranean Basin. Goat farming systems have not received significant investments due to their low required management cost and to the adaptation capacity of goats to harsh environments [4,5,6]. Due to goats having high metabolic efficiency and behavioral mechanisms, they are the livelihoods of poor farmers; they provide tangible (e.g., milk, meat, and manure) and intangible (e.g., savings and cultural services) benefits to mountainous societies [7,8]. 

Previous researches have investigated and detailed the diet composition of goats in Mediterranean forest rangelands [9,10,11]. The available studies on the nutritive value of some browse species were mainly conducted in the northern [6,12] and eastern Mediterranean countries [13,14]. In the Southside of the Mediterranean forest, most of the studies [15,16] focused solely on a few lists of plant species (less than ten). Specific parts of these plants were separately analyzed (leaves, stems, and twigs) from shrub species and collected only for one period or throughout their vegetative cycle. Nevertheless, these findings did not consider the actually consumed parts of the plant at grazing time. Furthermore, the nutritive value of browsed plant species by goats has been unexplored on forest rangelands of the Southern Mediterranean of northern Morocco.

Moreover, forage quality is characterized by seasonal variations that could affect plant selection by grazing animals and, thus, diet quality and quantity and animal performance. However, there are differences in the degree of these variations depending on each regional climate and vegetation types [17,18,19]. Extensive grazing goat production systems in northern Morocco are affected by annual dry periods, resulting in reduced animal performance, and farm profitability [7,20]. The changes in chemical composition and digestibility of plant species with grazing seasons during two consecutive years have not been investigated previously.

In northern Morocco, the existing forest vegetation, mountainous topography, and animal adaptation explain the predominance of grazing goats in forest rangelands [20]. In this area, extensive goat farming plays an important socioeconomic role and contributes from (approximately) 68% to 100% of farmer incomes [21]. Therefore, this study was carried out to follow the temporal evolution in the chemical composition, in vitro digestibility, and metabolizable energy (ME) of each plant species selected by goats in the Southern Mediterranean forest rangeland of northern Morocco over three grazing seasons of two consecutive years.

## 2. Materials and Methods

### 2.1. Description of the Sampling Area

This research was conducted in a Southern Mediterranean forest rangeland of the Western Rif (35°14′ N; 5°30′ W; 300 to 520 m a.s.l), located in northern Morocco. The climate of the region is influenced by the Atlantic Ocean, dominated by Mediterranean humid to sub-humid conditions (dry in summer and wet in winter). The site was studied for two consecutive years under contrasting climatic conditions, with 270- and 755-mm rainfalls in 2016 and 2017, respectively. The mean annual precipitation was estimated to 700 mm, with a daily temperature range of 3–14 °C (minimum) and 18–38 °C (maximum) [11]. Based on meteorological data of this last two decades, the year 2016 could be considered as dry and 2017 as a wet year. The study area is mountainous and characterized by relatively rugged topography. This forest pasture is covered mainly with shrub strata resulting from oak forest degradation. The high formation includes *Quercus ilex* L. and *Quercus suber* L. associated with shrublands dominated by *Arbutus unedo* L., *Cistus crispus* L., *Cistus monspeliensis* L., and *Erica arborea* L. [22,23].

### 2.2. Source of Forage Samples

The study area was covered by heterogeneous vegetation composed mainly of three distinct groups of plant species: shrubs (*A. unedo* L., *Calicotome villosa* (Poir.) Link, *Cistus* spp. (inclusive of *C. crispus* L., *C. monspeliensis* L., and *C. salviifolius* L.), *E. arborea* L., *Lavandula stoechas* L., *Myrtus communis* L., *Phillyrea media* L., *Pistacia lentiscus* L., and *Rubus ulmifolius* Schott.), trees (*Quercus* spp. (inclusive of *Q. canariensis* L., *Q. ilex* L., and *Q. suber* L.), and *Olea europaea* var. *sylvestris* (Mill) Lehr), and herbaceous (mainly *Anthemis cotula* L., *Brachypodium distachyon* L., *Bromus rigidus* Roth, *Calamintha nepeta* (L.) Kuntze, *Cynodon dactylon* (L.) Pers., *Eryngium tricuspidatum* L., *Lythrum junceum* Banks and Sol., *Rumex bucephalophorus* L.). According to Chebli et al. [11], these plant species are listed as the main dietary components of goats in Southern Mediterranean forest rangelands. Grazing in the forest rangelands of northern Morocco is practiced only over three seasons (spring, summer, and autumn). For the winter, pasture access is very limited; goats do not browse in forest pastures and graze only in fallow land around the goat shed, which explains the exclusion of this season from the study. The present research studied the chemical composition, in vitro digestibility, and metabolizable energy of all browsed species by goats. Samples were collected by hand-plucked simulation of each ingested part of the plant species similar to those consumed by goats. Diet composition and hand-plucked simulation are briefly summarized here and described fully in Chebli et al. [11]. The study concerned the botanical composition of each consumed part of plant by goats. The sampling was undertaken in the last month of each studied season (May, August, and November). Representative hand-plucked samples per plant species (a mixture of leaves and green tender stems) and herbaceous, similar to those consumed by goats, were imitated seasonally. For the thorny species, we used scissors to clip the selected parts. For herbaceous species, they were mixed into a single group because of difficulty to identify all ingested species by goats during grazing and their low selectivity. For shrubs and trees, the samples were harvested per species in special bags, with three replications, and transported to the laboratory for analysis.

### 2.3. Laboratory Analysis

Chemical analyses and in vitro digestibility studies were performed on three independent samples of the hand-plucked forage of each ingested plant species by goats during each grazing season of two consecutive years.

#### 2.3.1. Chemical Analysis

Collected samples were dried at 40 °C in a ventilated oven to minimize changes in tannins content and activity until reaching constant weight [24], and then milled with a sieve mesh size of 1 mm for analysis. Dry matter (DM), organic matter (OM), crude protein (CP), and ether extract (EE) were analyzed according to the Association of Official Analytical Chemists [25]. The neutral detergent fiber (NDF) was estimated using the Mertens [26] method with α-amylase and sodium sulfite. Acid detergent fiber (ADF) was determined according to method 973.18 of AOAC [27]. Acid detergent lignin (ADL) was determined by the solubilization of cellulose with sulfuric acid, according to Robertson and Van Soest [28]. All fiber extractions were performed using ANKOM 200 Fiber Analyzer® (ANKOM Technology, Fairport, NY, USA). The NDF, ADF, and ADL values were expressed inclusive of residual ash. Condensed tannins (CT) were predicted by Porter et al. [29] method using butanol-HCl, and ferric reagents.

#### 2.3.2. In Vitro Digestibility and Metabolizable Energy

In vitro dry matter (IVDMD) and organic matter (IVOMD) digestibility were performed using DAISYII Incubator® (ANKOM Technology, Fairport, NY, USA) as described by Mabjeesh et al. [30]. This device is essentially based on the in vivo simulation of digestion [31]. The rumen liquor for incubation was collected from five goats at a communal slaughterhouse, as described by El Otmani et al. [32]. These goats grazed in similar forest rangeland of the study area. The collected ruminal fluid was maintained in a thermos at 39 °C to keep rumen microflora alive. A weight of 0.5 g of each sample was placed in ANKOM filter bags (F57) and was put in jars (24 bags/jar). The inoculum, mixture containing 4/5 volume of artificial saliva, and 1/5 of rumen liquor was added in jars and incubated at 39.5 °C for 48 h. IVDMD and IVOMD were estimated by quantifying residuals DM and OM comparing to incubated initial quantities.

The metabolizable energy (ME; MJ/kg DM) of each consumed plant species was calculated using the equation [27]:

ME = 0.17 × DMD – 2,(1)

where DMD is the dry matter digestibility in percentage.

### 2.4. Statistical Analysis

Data were analyzed using SAS software® (SAS Inst. Cary, NC, USA). Chemical composition, digestibility, and ME of each plant species (*n* = 15) and herbaceous were analyzed using a general linear model (GLM) procedure of SAS in a factorial structure. Data were compared between seasons (i.e., spring, summer, and autumn), years (i.e., 2016 and 2017), and their interactions. Simple correlation analysis was used to establish the relationships between the chemical composition, IVOMD, and ME. The correlation plot was obtained by utilizing the “*corrplot*” library in the R-package [33]. For all analyses, the significance level was declared at *p* < 0.05. In case of significant effect, means were compared using the Tukey’s test.

## 3. Results

The chemical composition, IVOMD, and ME of the browsed plant species by goats at different sampling seasons and years are given in Table 1 (shrubs) and Table 2 (trees and herbaceous). Overall, these parameters of shrubs (*n* = 11), trees (*n* = 4), and herbaceous species varied seasonally in each studied year.

Across shrub species, all of them presented a higher DM content in summer, except for *C. salviifoluis* and *M. communis,* with a higher DM content in spring, and for *R. ulmiformis*, with a higher DM content (also) in autumn. The higher water content was observed either in spring (for five shrubs) or autumn (for six shrubs). This parameter was significantly affected by both studied factors (season and year) except for *C. crispus,* which was not affected by year. Their effects on OM of the studied shrub species were variable, *A. unedo*, *E. arborea, L. stoechas, M. communis,* and *P. media,* having the same OM throughout the year. The CP content varied significantly among seasons of both years (*p* < 0.05), except for the season effect of the dry year (2016) on *C. villosa* and the season effect of the wet year (2017) on *A. unedo, C. crispus, C. salviifoluis*, *E. arborea,* and *P. media* (*p* > 0.05). During both years, the highest and lowest CP concentrations were recorded in *C. villosa* (about 240 g/kg DM in the autumn) and *A. unedo* (about 60 g/kg DM in summer), respectively. The CT content ranged from 1.97 g/kg DM (summer 2017) in *C. villosa* to 191 g/kg DM (summer 2016) in *P. lentiscus*. The EE content ranged from 15.8 g/kg DM in *C. crispus*, to 90–101 g/kg DM in *C. monspeliensis* (summer and autumn) and *E. arborea* (spring and summer). The highest NDF and ADF levels of both years were observed in *C. villosa*, with 629 and 482 g/kg DM, respectively. Overall, the ADL contents showed a significant increase from spring to summer–autumn (except for *P. media*) with a range from 62.3 g/kg DM in *R. ulmifolius* (2016) to 324 g/kg DM in *E. arborea* (2017). All studied shrub species presented a higher IVOMD in spring of both consecutive years, except for *R. ulmifolius* and *M. communis*, with the highest IVOMD in summer–autumn and summer, respectively. The lower IVOMD were found in summer (for five shrubs) or autumn (for two shrubs), or there was no significant difference between summer and autumn (for two shrubs). The ME results showed the same trend as the IVOMD. The highest ME content was observed in *L. stoechas* (about 10 MJ/kg DM) browsed during spring and the lowest one (about 4.5 MJ/kg DM) in *C. villosa* (autumn) and *E. arborea* (summer) during both dry and wet years. The most notable changes due to advancing maturity were found in the CP, CT, ADL, and ME contents. Generally, the CP and ME contents decreased, and CT and ADL contents increased during spring to summer–autumn of both years.

Across trees species and for the two years, DM content was higher in summer and lower in spring, except for *O. europaea*, where it was the opposite. The DM content in autumn was the same as in spring for *Q. suber*, the same as in summer for *O. europaea* and *Q. ilex*, and significantly different from the two other seasons for *Q. canariensis*. Each oak tree species showed no variation of its OM content during a year, except for *Q. suber* in 2016, which presented a decrease over time. *Q. ilex* recorded the higher CP concentration in spring of the wet and dry years (99.7 and 114 g/kg DM, respectively). *Q. suber* had the highest, and *O. europaea* the lowest CT content during all studied seasons. Among tree species, the oak trees showed low EE content (about 24 g/kg DM). The high EE content was recorded in *O. europaea* during the summer of the wet year (131 g/kg DM). The highest NDF content was recorded for *Q. suber* and *Q. ilex* (about 550 g/kg DM), and the lowest for *O. europaea* (about 410 g/kg DM). The highest and lowest ADL levels were observed during autumn and spring of the wet year in *Q. ilex* (about 190 g/kg DM) and in *Q. canariensis* (103 g/kg DM), respectively. *Quercus* spp. had a high IVOMD significantly during spring and a low one in summer. The IVOMD of *O. europaea* was similar in all studied seasons during the dry year. Nevertheless, this similarity was not observed in the wet year, with a slight increase over time. The ME levels of the studied tree species varied slightly, being particularly low in *Quercus* spp. (about 6 MJ/kg DM) during the summer of the dry and wet years and highest in *Q. canariensis* (8.8 MJ/kg DM) during the two springs.

Comparatively to the two other groups, herbaceous also had a higher DM content in summer and a lower content in spring. The OM content was variable according to the year and season. It was higher in the spring and similar and lower in the other seasons. The CP concentrations recorded the highest value in the spring of 2016 and 2017 (156 and 142 g/kg DM, respectively; *p* < 0.001). The CT contents recorded the highest values during the summer of 2016 (4.17 g/kg DM; *p* < 0.05) but were similar among seasons of 2017 (with about 2.7 g/kg DM; *p* = 0.116). The NDF concentrations were similar among seasons of both years (*p* > 0.05). The ADF content was higher in summer than spring and autumn of both years (*p* < 0.01). The ADL concentrations were similar among seasons of 2016 (about 70 g/kg DM; *p* = 0.265) but increased in 2017 from spring to summer (*p* < 0.05). The IVOMD and ME contents were higher in spring than in autumn and summer (*p* < 0.001). Overall, the highest CP and ME contents were recorded in the herbaceous and the lowest in shrub and tree species. An opposite trend was recorded for CT and ADL levels.

**Figure 1:**
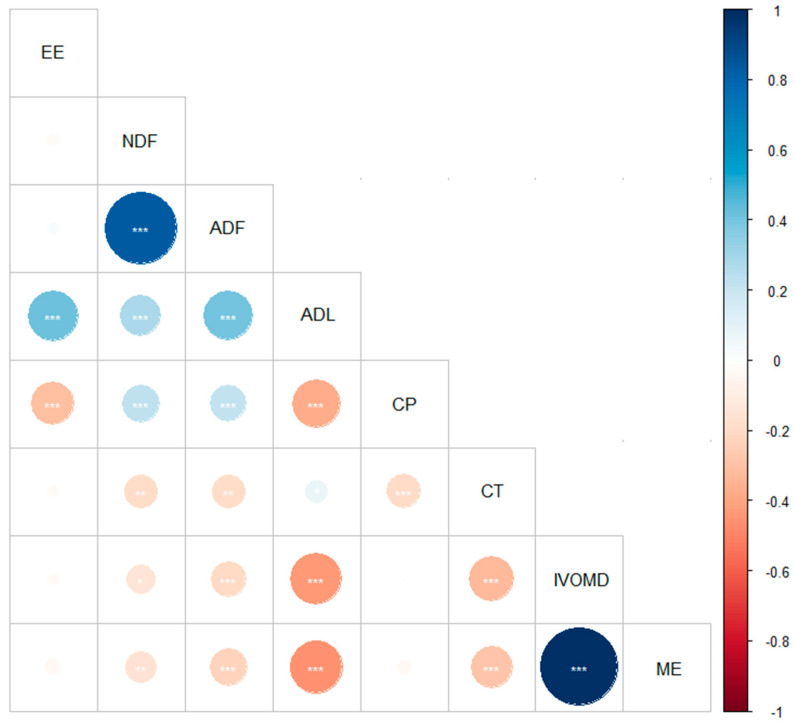
Correlation plot between the chemical composition, IVOMD, and ME from forage species browsed by goats. Positive and negative correlation coefficients are displayed in blue and brown scale, respectively. EE, ether extract; NDF, neutral detergent fiber; ADF, acid detergent fiber; ADL, lignin; CP, crude protein; CT, condensed tannins; IVOMD, in vitro organic matter digestibility; ME, metabolizable energy. Significance level (*** < 0.001, ** < 0.01, and * < 0.05).

The correlation values among the chemical composition, IVOMD, and ME from the studied forage species are presented in Figure 1. The CP showed a negative correlation with ADL, CT, and EE (*p* < 0.001). The ME was strongly correlated with IVOMD (*p* < 0.001). The NDF, ADF, and ADL contents were positively correlated with each other (*p* < 0.001). A negative correlation was observed between IVOMD and ME with CT, ADL, and ADF (*p* < 0.001), and with NDF (*p* < 0.05).

## 4. Discussion

The aim of this study was to assess the nutritive value of the plant species browsed by goats and their variations throughout three grazing seasons of two years. These years appeared very contrasted regarding the mean annual rainfall, with a dry year in 2016 and a wet one in 2017. According to Papachristou et al. [34], the bulk of the grazing goats’ diet includes few ligneous and herbaceous species, representing less than ten species. Ligneous species *A. unedo*, *C. villosa*, *E. arborea*, *M. communis*, *P. lentiscus*, and *Q. suber* are considered the most widespread species in the Southern Mediterranean rangelands [15,23]. As observed and described by Chebli et al. [11], plant species analyzed herein represent the all-selected diet by grazing goats in Southern Mediterranean forest rangelands. During spring of 2016, the contribution of *C. monspeliensis* (28.8%), *C. crispus* (19.8%), and *C. salviifolius* (17.6%) was the highest followed by *L. stoechas* (17.3%) and herbaceous (7%). These species contributed lowly to the diet during autumn and summer (< 3%). In the autumn and summer, the diet proportion of *Quercus* spp. (3–20%), *M. communis* (14–19.4%), *P. lentiscus* (8–13%)*, A. unedo* (11–13%), *E. arborea* (9.5–11%), and *O. europaea* (2–7%) was largely significant. During spring of 2017, the contribution of *C. crispus* was significantly increased by 42% with the decreased rate of *C. salviifolius* and *L. stoechas* by 10 and 15%, respectively. In the autumn, the greatest increase in contribution to the diet was observed for *O. europaea* followed by *P. lentiscus*, and E. *arborea*. The opposite trend was observed with the diet proportion of *Q. canariensis* and *C. villosa*. In the summer, the contribution of *P. lentiscus* and *P. media* increased by 93 and 17%, respectively. On the other hand, diet contribution of *A. unedo* and *E. arborea* decreased by 35 and 17%, respectively. On average, the diet of the goats was largely composed of shrubs (64–90%) and trees (2–35%). However, the contribution of herbaceous did not exceed 8%. The contribution of trees to the diet during spring dropped from 30.3 to 3.7% and from 29.0 to 2.2% in 2016 and 2017, respectively. The diet proportion of *R. ulmifolius* varied from 0.01 to 3.4% [11].

The nutritive value of the hand-plucked samples, corresponding to the most tender part of the plant, appears to reflect the quality of the diet consumed by grazing goats [6]. For this study, the browsed parts of the plant by goats were analyzed, which represent a mixture of leaves, stems, and twigs.

The chemical traits of browsed species were extremely wide, which are in accordance with previous studies conducted in northern and eastern Mediterranean forest rangelands [6,12,13,14], deciduous tropical forest [35], South African rangeland [36]. In northwestern Italy, Ravetto Enri et al. [37] reported the relevant effect of the vegetative season on chemical composition and in vitro true digestibility of four tree species selected by goats. These wide variations on the nutritional proprieties of plant species could be explained by soil fertility [15,38], environmental conditions, and stage of growth or age [14,39]. 

The observed mean CP level found in this study varied from 60 to 240 g/kg DM. Most of the analyzed plant species present high levels of CP than the minimum level of 70–80 g/kg DM required by microorganisms for optimum rumen functioning and feed intake in ruminant livestock [40]; a lower CP content affects negatively feed intake and digestibility [41]. In the present study, the low CP content was particularly recorded in *A. unedo* (from 52.7 to 70.7 g/Kg DM). In the northwest of Tunisia, a value of 55 g/kg DM in *A. unedo,* collected in March 1998 from the uplands of Taaref, was reported [15], which is in the range of the current results. The high proportions of mature leaves and twigs in the samples could explain the low CP level in some plant species, such as *A. unedo*. Overall, the average CP level was higher during spring 2016 in all species because plants contain the maximum CP content during the vegetative stage [42]. The decrease of this parameter in the summer agrees with the literature [19,43] because CP drops with the physiological maturity stage of the plant [44], which explains the negative correlation of CP with ADL and EE that increase with plant maturity as found by Ammar et al. [12] in some Spanish shrub species. As expected, the CP content was higher in *C. villosa* as it is a leguminous plant. According to Kokten et al. [14], leaves of *C. villosa* could be used as protein supplements for livestock since their CP contents are high compared to the other Mediterranean shrubs. The high protein level in *C. villosa* could be attributed to the ability of this plant to fix atmospheric nitrogen thanks to rhizobia associated with their nodules [12,16]. Overall, the older leaves contained less CP and more fiber than the young and tender part of the selected plant species. This statement is in consistent with other studies [6,14]. In terms of CP content, many of the woody species cover the daily maintenance requirements of grazing goat but not for milk or meat production needs, which is above 130 g/kg DM. In another environment (hills of Nepal), Khanal and Subba [45] reported a good nutritional value of leaves from most of the tree fodder species, with a minimum CP of 110 g/kg DM. 

Generally, trees had a higher EE content during summer, which coincides with the maturity stage of these groups of plant species. Indeed, plant species had a higher fat content (EE) in the late physiological stage that increases with maturity [12].

According to the species and sampling season, the NDF, ADF, and ADL contents in ligneous species varied from 242 to 629 g/kg, 186 to 482 g/kg, and 70 to 322 g/kg, respectively. However, herbaceous recorded the lower lignin content (64.3–89 g/kg). Overall, these contents significantly increased by advancing maturity. The results are in line with the findings of several authors [14,16,46], who indicated that cell wall content (NDF, ADF, and ADL) augmented with maturity (cell wall lignification). All analyzed samples recorded higher ADL levels during the spring of the dry year compared to the wet year. This higher concentration during the dry year could reflect the response of plant species to water stress (rarity rains), which is associated with the increased level of tannins. Khanal and Subba [45] reported a high ADL content in most of the fodder trees in the hills of Nepal, with values more than 100 g/kg DM. High fiber content, and lignin especially, means low free-nitrogen extract and soluble carbohydrates contents, which explain the observed negative correlations of fibers with IVOMD and ME. Ammar et al. [47] reported an increase of fiber content in parallel with a decrease of in vitro digestibility, with the maturity of mountain grasses, which confirm the negative correlation between fibers and digestibility.

The observed CT concentrations varied from 2.3 to 184 g/kg DM, showing significantly different and slightly higher values than those obtained by several authors [12,48] with shrub leaves from Northern Spain. These variations could be due in part to the difference in analysis methods. Moreover, it could be owing to the stage of growth and the sampled parts of the plants (leaves, stems, and twigs), to the season and to the nature of the sampling site [49,50]. In addition, the current study concerns the analysis of different parts of the plant selected by goats, not only their leaves.

A CT concentration of 20–45 g/kg DM has a negative effect on protein digestibility and proteolytic bacteria [51], and a concentration above 55 g/kg DM reduces the voluntary feed intake of grazing ruminants [52,53]. Thus, except for herbaceous (2.20–4.17 g/kg DM), *C. villosa*, *L. stoechas*, *P. media*, and *O. europaea*, all pastoral species had a CT content higher than this maximum level. However, even with a high CT content, the shrubs were highly consumed in spring and autumn [11]. These findings are consistent with Fomum et al. [36] and Mkhize et al. [54], who reported no correlation between CT and feed intake in goats. It could be explained by the ability of goat to balance their diet and dilute secondary compounds by consuming a mixture of plant species [55,56]. Moreover, grazing animals exposed to high CT feed could excrete more saliva richer in proline-rich proteins that has the ability to bind with CT to neutralize it [52,54]. Nevertheless, goats have a specificity compared to other ruminants that their ruminal microbiota is able to valorize feed with low nutritional values due to their cellulolytic bacteria and the tanninase activity [57]. Min et al. [51], reported that high CT concentrations reduced digestibility, which could explain the negative correlation between CT and IVOMD.

Bartolomé et al. [58], who studied the quality of forest resources in the undergrowth of the pine forests of Mallorca (Spain), reported that species, such as *P. lentiscus, C. monspeliensis*, and *A. unedo*, could be interesting as feed for goats, as they show protein levels above the minimum of maintenance, and at the same time, high digestibility. Nevertheless, they present a high content of secondary compounds, such as tannins.

In the present study, the higher values of digestibility, mainly observed in spring, are attributed to its negative correlation with ADF and ADL. Ammar et al. [12], also reported a negative correlation between fibers and digestibility of browsed leaves. Most of the pastoral species had low digestibility and, consequently, a low energy content (IVOMD < 550 g/kg; ME < 8 MJ/kg DM), except for herbaceous and some shrubs (*A. unedo* and *Cistus* spp.), as their values varied from medium (IVOMD: 550–700 g/kg; ME: 8–10 MJ/kg DM) to higher nutritional values (IVOMD > 700 g/kg; ME > 10 MJ/kg DM), especially during spring [59]. As reported by Paton [60], ME depends mainly on IVOMD, which could explain their high positive correlation. Overall, goats select species with high CP and digestibility and low fiber content basis, in accordance with the literature [34].

## 5. Conclusions

This study provides a valuable and useful database on the temporal variations in chemical composition, in vitro digestibility, and ME of the main plant species browsed by goats in the Southern Mediterranean forest rangeland. Most of these plant species showed considerable variation among grazing season. In general, the nutritive value of plant species was highest in spring, and then steadily decreased through the summer. In autumn, the nutritive value decreased, remained the same, or increased compared to summer. Most of the selected plant species presented high levels of CP than the minimum required levels for maintenance needs. All analyzed samples recorded higher lignin levels during the spring of the dry year compared to the wet year. Except for herbaceous, *C. villosa*, *L. stoechas*, *P. media*, and *O. europaea*, all analyzed species had a CT content higher than this maximum level. The high values of digestibility in spring are attributed to its negative correlation with ADF and lignin. Owing to the morphological and physiological differences between the consumed plant species, changes in chemical compositions and in vitro digestibility could be expected. Consequently, goats are forced to adapt their browsing behavior to the low-quality vegetation, typical of the Mediterranean forest. The results could be used as indicators to assess the nutritional value of the goat diets in forest rangelands throughout the year to help the grazing management strategy, and/or to eventually supplement the goats adequately, to prevent the low farmer incomes due to the animal performance decrease.

Future work would allow knowing if the practiced grazing systems in the Southern Mediterranean region could guarantee the seasonal dietary requirements of grazing goats, in terms of energy and protein, taking into account the physical and physiological conditions of grazing goats.

## Figures and Tables

**Table 1:** Chemical composition (g/kg DM), IVOMD (g/kg), and ME (MJ/kg DM) of shrub species (*n* = 11) browsed by goats in Southern Mediterranean forest rangeland of northern Morocco during two contrasting years.

Item	2016 (Dry Year)					2017 (Wet Year)					SEM	*p*-Value (2016–2017)		
	Spring	Summer	Autumn	SEM	*p*-Value	Spring	Summer	Autumn	SEM	*p*-Value		S	Y	Y × S
*Arbutus unedo*														
DM	576 ^b^	660 ^a^	445 ^c^	31.6	<0.001	550 ^b^	647 ^a^	427 ^c^	32.3	<0.001	22	<0.001	0.035	0.828
OM	962	964	955	4.7	0.768	974	974	968	3.72	0.775	3.23	0.6	0.116	0.99
CP	69.8 ^a^	52.7 ^b^	60.3 ^ab^	2.79	0.009	60	67.3	70.7	2.24	0.13	1.84	0.156	0.054	0.003
CT	91.4 ^b^	112 ^ab^	121 ^a^	5.1	0.014	83.7 ^b^	101 ^ab^	110 ^a^	4.56	0.018	3.53	<0.001	0.028	0.926
EE	70.3 ^b^	91.3 ^a^	67.7 ^b^	4.11	0.005	73.3 ^b^	96.6 ^a^	72.3 ^b^	4.27	0.003	2.93	<0.001	0.138	0.936
NDF	354 ^b^	485 ^a^	482 ^a^	23.5	0.004	344 ^b^	491 ^a^	488 ^a^	26.1	0.003	17	<0.001	0.972	0.884
ADF	257 ^c^	324 ^b^	363 ^a^	16	<0.001	243 ^c^	346 ^b^	377 ^a^	20.4	<0.001	12.6	<0.001	0.203	0.056
ADL	110 ^b^	172 ^a^	185 ^a^	12.7	0.004	105 ^b^	191 ^a^	197 ^a^	15.7	0.001	9.82	<0.001	0.324	0.538
IVOMD	603 ^a^	506 ^b^	387 ^c^	31.5	<0.001	617 ^a^	512 ^b^	405 ^c^	30.8	<0.001	21.4	<0.001	0.037	0.637
ME	9.11 ^a^	7.34 ^b^	5.13 ^c^	0.582	<0.001	9.23 ^a^	7.56 ^b^	5.20 ^c^	0.591	<0.001	0.403	<0.001	0.372	0.899
*Calicotome villosa*														
DM	228 ^c^	487 ^a^	366 ^b^	37.6	<0.001	215 ^c^	471 ^a^	326 ^b^	37.1	<0.001	25.7	<0.001	0.002	0.138
OM	927 ^b^	984 ^a^	950 ^ab^	10.1	0.038	937 ^b^	988 ^a^	961 ^ab^	9.33	0.044	6.75	0.002	0.383	0.95
CP	175	190	232	10.9	0.059	161 ^b^	201 ^ab^	238 ^a^	12.9	0.016	8.18	0.001	0.944	0.603
CT	3.81	2.43	2.97	0.439	0.492	2.87	1.97	2.09	0.202	0.133	0.252	0.178	0.137	0.905
EE	31.3 ^a^	27.0 ^a^	22.0 ^b^	1.44	0.002	34.3 ^a^	30.3 ^b^	30.7 ^ab^	0.777	0.033	0.999	<0.001	<0.001	0.019
NDF	520 ^b^	619 ^a^	593 ^a^	15.3	<0.001	511 ^b^	629 ^a^	601 ^a^	18.1	<0.001	11.5	<0.001	0.632	0.425
ADF	417 ^b^	464 ^a^	417 ^b^	8.63	0.007	406 ^b^	482 ^a^	429 ^b^	12	0.002	7.22	<0.001	0.366	0.212
ADL	95.7 ^b^	124 ^a^	117 ^a^	4.65	0.004	94.7 ^c^	138 ^a^	128 ^b^	7.08	<0.001	4.19	<0.001	0.017	0.028
IVOMD	545 ^a^	439 ^b^	351 ^c^	28.4	<0.001	554 ^a^	443 ^b^	362 ^c^	28.1	<0.001	19.4	<0.001	0.239	0.906
ME	7.74 ^a^	6.17 ^b^	4.42 ^c^	0.485	<0.001	7.89 ^a^	6.16 ^b^	4.49 ^c^	0.499	<0.001	0.338	<0.001	0.6077	0.889
*Cistus crispus*														
DM	414 ^b^	528 ^a^	344 ^c^	27	<0.001	399 ^b^	514 ^a^	332 ^c^	26.9	<0.001	18.6	<0.001	0.064	0.983
OM	945 ^a^	946 ^a^	914 ^b^	6.56	0.049	963 ^a^	951 ^ab^	927 ^b^	6.56	0.034	4.73	0.002	0.083	0.695
CP	113 ^a^	60.3 ^b^	76.0 ^b^	8.71	0.009	99.7	79.3	85.3	5.05	0.266	4.92	0.002	0.451	0.166
CT	15.1 ^b^	65.0 ^a^	61.7 ^a^	8.2	<0.001	13.7 ^b^	61.3 ^a^	54.0 ^a^	7.51	<0.001	5.42	<0.001	0.083	0.537
EE	15.8 ^b^	21.8 ^a^	17.9 ^ab^	0.986	0.011	19	23.3	20.3	0.873	0.098	0.7	0.001	0.018	0.758
NDF	309 ^b^	242 ^c^	384 ^a^	20.8	<0.001	305 ^b^	256 ^c^	393 ^a^	20.3	<0.001	14.1	<0.001	0.318	0.43
ADF	266 ^a^	207 ^b^	252 ^a^	10.4	0.02	251	226	265	7.46	0.076	6.25	0.002	0.529	0.257
ADL	103 ^b^	184 ^a^	170 ^a^	12.9	<0.001	93.0 ^b^	195 ^a^	184 ^a^	16.4	<0.001	10.1	<0.001	0.261	0.101
IVOMD	642 ^a^	408 ^c^	488 ^b^	34.3	<0.001	652 ^a^	412 ^c^	498 ^b^	35.2	<0.001	23.9	<0.001	0.039	0.676
ME	9.63 ^a^	5.59 ^c^	7.12 ^b^	0.589	<0.001	9.76 ^a^	5.71 ^c^	7.20 ^b^	0.592	<0.001	0.405	<0.001	0.059	0.891
*Cistus monspeliensis*														
DM	585 ^b^	698 ^a^	379 ^c^	46.8	<0.001	573 ^b^	676 ^a^	366 ^c^	45.8	<0.001	31.8	<0.001	0.011	0.717
OM	905 ^c^	952 ^a^	930 ^b^	6.94	<0.001	918 ^c^	966 ^a^	947 ^b^	7.32	<0.001	5.21	<0.001	<0.001	0.832
CP	98.4 ^a^	84.7 ^ab^	66.3 ^b^	5.36	0.015	88.0 ^ab^	98.7 ^a^	73.7 ^b^	4.39	0.032	3.39	0.001	0.405	0.088
CT	46.0 ^c^	65.6 ^b^	78.0 ^a^	4.82	<0.001	40.7 ^b^	54.7 ^a^	65.7 ^a^	3.93	0.004	3.23	<0.001	0.001	0.439
EE	56.4 ^b^	90.6 ^a^	96.0 ^a^	6.39	<0.001	60.6 ^b^	98.6 ^a^	101 ^a^	6.78	<0.001	4.57	<0.001	0.061	0.845
NDF	388 ^c^	492 ^a^	434 ^b^	15.2	<0.001	377 ^c^	503 ^a^	440 ^b^	18.3	<0.001	11.6	<0.001	0.588	0.062
ADF	220 ^c^	255 ^b^	314 ^a^	13.8	<0.001	206 ^c^	274 ^b^	325 ^a^	17.6	<0.001	10.9	<0.001	0.23	0.028
ADL	172 ^b^	176 ^b^	205 ^a^	5.81	0.007	162 ^c^	187 ^b^	221 ^a^	8.72	<0.001	5.13	<0.001	0.164	0.04
IVOMD	592 ^a^	407 ^c^	489 ^b^	26.8	<0.001	601 ^a^	412 ^c^	501 ^b^	27.4	<0.001	18.6	<0.001	0.012	0.617
ME	8.27 ^a^	5.39 ^c^	6.72 ^b^	0.417	<0.001	8.46 ^a^	5.49 ^c^	6.77 ^b^	0.431	<0.001	0.291	<0.001	0.053	0.562
*Cistus salviifolius*														
DM	488 ^a^	441 ^b^	366 ^c^	18	<.0001	477 ^a^	424 ^b^	353 ^c^	18.2	<0.001	12.5	<0.001	0.019	0.913
OM	876 ^b^	854 ^b^	906 ^a^	8	0.001	890 ^b^	861 ^c^	921 ^a^	9.24	0.001	6.1	<0.001	0.021	0.714
CP	108 ^a^	80.7 ^b^	70.7 ^b^	5.73	<0.001	95.7	94	83	2.68	0.091	3.11	<0.001	0.107	0.002
CT	25.0 ^c^	78.0 ^a^	49.7 ^b^	7.96	<0.001	21.0 ^c^	61.0 ^a^	41.7 ^b^	5.91	<0.001	4.95	<0.001	0.006	0.215
EE	23.3 ^b^	50.3 ^a^	40.6 ^a^	4.23	0.002	27.3 ^c^	63.6 ^a^	46.3 ^b^	5.42	<0.001	3.46	<0.001	0.007	0.274
NDF	417 ^c^	506 ^a^	485 ^b^	13.6	<0.001	406 ^c^	515 ^a^	496 ^b^	16.9	<0.001	10.5	<0.001	0.434	0.025
ADF	252 ^c^	341 ^a^	291 ^b^	13.23	<0.001	239 ^c^	360 ^a^	303 ^b^	17.7	<0.001	10.7	<0.001	0.282	0.071
ADL	154 ^c^	228 ^a^	206 ^b^	11.1	<0.001	142 ^b^	238 ^a^	224 ^a^	15.2	<0.001	9.14	<0.001	0.08	0.003
IVOMD	602 ^a^	439 ^b^	440 ^b^	27.5	<0.001	611 ^a^	445 ^b^	455 ^b^	27.2	<0.001	18.8	<0.001	0.181	0.877
ME	8.47 ^a^	5.91 ^c^	6.32 ^b^	0.398	<0.001	8.60 ^a^	5.91 ^c^	6.38 ^b^	0.416	<0.001	0.279	<0.001	0.302	0.725
*Erica arborea*														
DM	571 ^b^	650 ^a^	500 ^c^	21.8	<0.001	551 ^b^	634 ^a^	475 ^c^	23.1	<0.001	15.6	<0.001	0.001	0.711
OM	947	955	965	4.69	0.331	960	967	977	4.15	0.24	3.41	0.083	0.051	0.999
CP	88.7 ^a^	53.7 ^c^	69.7 ^b^	5.38	0.002	73.7	65	77.3	2.66	0.146	2.92	<0.001	0.673	0.009
CT	108	107	119	2.78	0.143	100	91.7	108	3.21	0.111	2.48	0.019	0.006	0.674
EE	96.3 ^a^	90.0 ^a^	46.6 ^b^	7.89	<0.001	99.6 ^a^	93.6 ^a^	57.0 ^b^	6.77	<0.001	5.09	<0.001	0.012	0.294
NDF	439 ^c^	531 ^b^	578 ^a^	21	<0.001	428 ^c^	544 ^b^	586 ^a^	24	<0.001	15.4	<0.001	0.611	0.323
ADF	341 ^c^	399 ^b^	445 ^a^	15.6	<0.001	328 ^c^	414 ^b^	458 ^a^	19.5	<0.001	12.1	<0.001	0.448	0.178
ADL	217 ^b^	307 ^a^	311 ^a^	15.4	<0.001	207 ^b^	320 ^a^	324 ^a^	19.3	<0.001	12	<0.001	0.042	0.002
IVOMD	479 ^a^	343 ^c^	407 ^b^	19.9	<0.001	486 ^a^	348 ^c^	417 ^b^	20.3	<0.001	13.8	<0.001	0.257	0.948
ME	6.62 ^a^	4.58 ^c^	5.79 ^b^	0.299	<0.001	6.77 ^a^	4.41 ^c^	5.86 ^b^	0.347	<0.001	0.222	<0.001	0.891	0.282
*Lavandula stoechas*														
DM	299 ^c^	475 ^a^	409 ^b^	25.7	<0.001	281 ^c^	459 ^a^	385 ^b^	25.8	<0.001	17.8	<0.001	<0.001	0.491
OM	944	939	916	6.24	0.133	953	954	930	6.25	0.217	4.56	0.031	0.116	0.936
CP	106 ^a^	83.0 ^b^	72.7 ^b^	5.09	<0.001	224 ^a^	96.7 ^b^	80.0 ^b^	2.81	0.014	2.83	<0.001	0.374	<0.001
CT	3.07	3.57	2.57	0.222	0.191	2.57	2.93	2.2	0.237	0.513	0.169	0.118	0.136	0.941
EE	90.0 ^a^	34.3 ^b^	33.0 ^b^	9.41	<0.001	96.6 ^a^	38.6 ^b^	42.3 ^b^	9.38	<0.001	6.5	<0.001	<0.001	0.112
NDF	418 ^c^	472 ^a^	446 ^b^	8.24	0.001	409 ^c^	486 ^a^	455 ^b^	11.5	<0.001	6.87	<0.001	0.341	0.11
ADF	253 ^b^	313 ^a^	298 ^a^	9.42	<0.001	238 ^b^	326 ^a^	313 ^a^	13.9	<0.001	8.16	<0.001	0.312	0.03
ADL	173 ^b^	208 ^a^	211 ^a^	6.23	<0.001	160 ^b^	215 ^a^	224 ^a^	10.1	<0.001	5.76	<0.001	0.264	0.002
IVOMD	698 ^a^	476 ^c^	512 ^b^	34.5	<0.001	704 ^a^	484 ^c^	522 ^b^	34	<0.001	23.5	<0.001	0.108	0.936
ME	10.2 ^a^	6.69 ^c^	7.29 ^b^	0.54	<0.001	10.3 ^a^	6.90 ^c^	7.40 ^b^	0.539	<0.001	0.371	<0.001	0.025	0.829
*Myrtus communis*														
DM	554 ^a^	531 ^b^	437 ^c^	18.1	<0.001	533 ^a^	515 ^a^	420 ^b^	17.7	<0.001	12.4	<0.001	<0.001	0.851
OM	952	939	948	3.79	0.428	962	953	959	3.31	0.571	2.81	0.245	0.045	0.959
CP	89.7 ^a^	72.3 ^b^	82.7 ^ab^	3.03	0.03	75.7 ^b^	84.7 ^ab^	90.7 ^a^	2.57	0.022	1.94	0.061	0.415	0.002
CT	96.0 ^b^	128 ^a^	115 ^a^	4.88	0.002	88.0 ^b^	116 ^a^	110 ^a^	4.47	0.002	3.37	<0.001	0.008	0.562
EE	42.6 ^a^	41.0 ^a^	24.0 ^b^	3.09	<0.001	46.3 ^a^	48.0 ^a^	27.6 ^b^	3.37	<0.001	2.29	<0.001	0.005	0.543
NDF	379 ^a^	362 ^ab^	336 ^b^	7.37	0.027	369	372	346	6.05	0.17	4.64	0.006	0.642	0.462
ADF	218	228	242	4.65	0.067	205 ^b^	242 ^a^	252 ^a^	7.76	0.003	4.41	<0.001	0.475	0.087
ADL	102	93	94.3	2.01	0.169	93.7 ^b^	108 ^a^	106 ^a^	2.55	0.015	1.74	0.571	0.019	0.003
IVOMD	493 ^b^	550 ^a^	485 ^b^	10.6	<0.001	500 ^b^	556 ^a^	504 ^b^	9.21	<0.001	6.94	<0.001	0.024	0.374
ME	7.24 ^b^	8.24 ^a^	7.16 ^b^	0.178	<0.001	7.41 ^b^	8.02 ^a^	7.28 ^b^	0.12	<0.001	0.104	<0.001	0.673	0.047
*Phillyrea media*														
DM	523 ^c^	612 ^a^	570 ^b^	13.1	<0.001	507 ^c^	598 ^a^	555 ^b^	13.5	<0.001	9.31	<0.001	0.004	0.974
OM	960	970	962	2.74	0.338	974	978	975	2.52	0.78	2.3	0.3	0.01	0.843
CP	109 ^a^	86.3 ^b^	81.0 ^b^	4.4	<0.001	96	97.3	92	1.48	0.351	2.28	<0.001	0.124	<0.001
CT	2.73	2.67	3.17	0.28	0.784	2.4	2.2	2.77	0.241	0.685	0.186	0.553	0.344	0.991
EE	23.6	25.6	28	1.22	0.401	28.6	28.6	31.6	1.47	0.692	1.04	0.337	0.076	0.918
NDF	399 ^b^	435 ^a^	424 ^ab^	6.79	0.047	387 ^b^	448 ^a^	429 ^a^	9.98	0.007	5.86	0.003	0.765	0.379
ADF	272 ^a^	259 ^ab^	250 ^b^	4.07	0.043	258 ^b^	272 ^a^	255 ^b^	2.92	0.005	2.44	0.006	0.651	0.011
ADL	171 ^a^	124 ^b^	119 ^b^	8.42	<0.001	159 ^a^	136 ^b^	134 ^b^	4.56	0.008	4.69	<0.001	0.126	0.007
IVOMD	515 ^a^	429 ^b^	413 ^b^	16.9	0.002	523 ^a^	435 ^b^	425 ^b^	16.7	0.003	11.6	<0.001	0.396	0.967
ME	7.29 ^a^	5.96 ^b^	5.82 ^b^	0.247	0.001	7.46 ^a^	5.76 ^b^	5.90 ^b^	0.285	<0.001	0.183	<0.001	0.889	0.504
*Pistacia lentiscus*														
DM	547 ^c^	622 ^a^	590 ^b^	11.1	<0.001	530 ^c^	607 ^a^	578 ^b^	11.5	<0.001	7.98	<0.001	0.006	0.882
OM	959 ^a^	927 ^b^	954 ^a^	5.25	0.001	973 ^a^	941 ^b^	964 ^a^	5.21	0.005	3.91	<0.001	0.001	0.79
CP	93.0 ^b^	91.7 ^b^	106 ^a^	2.41	0.003	78.0 ^b^	105 ^a^	113 ^a^	5.45	<0.001	2.9	<0.001	0.32	<0.001
CT	175	191	185	3.64	0.187	161 ^b^	177 ^a^	172 ^ab^	2.85	0.033	2.79	0.012	0.003	0.997
EE	27.3 ^a^	23.6 ^b^	23.3 ^b^	0.741	0.016	34	30	27.3	1.52	0.214	1.07	0.027	0.002	0.716
NDF	448 ^b^	483 ^a^	422 ^c^	8.98	<0.001	437 ^b^	493 ^a^	426 ^b^	10.4	<0.001	6.68	<0.001	0.591	0.012
ADF	284 ^a^	248 ^b^	270 ^ab^	5.95	0.014	268	263	284	4.33	0.109	3.61	0.006	0.363	0.037
ADL	118 ^b^	165 ^a^	168 ^a^	8.5	0.002	109 ^b^	178 ^a^	186 ^a^	12.57	<0.001	7.42	<0.001	0.132	0.069
IVOMD	505 ^a^	443 ^c^	471 ^b^	9.28	<0.001	508 ^a^	453 ^c^	483 ^b^	8.18	<0.001	6.09	<0.001	0.022	0.453
ME	7.21	6.6	6.74	0.121	0.069	7.37 ^a^	6.42 ^b^	6.83 ^ab^	0.159	0.018	0.097	0.001	0.876	0.567
*Rubus ulmifolius*														
DM	371 ^b^	409 ^a^	410 ^a^	6.69	<0.001	356 ^b^	394 ^a^	406 ^a^	8.43	0.008	5.42	<0.001	0.033	0.601
OM	908 ^b^	924 ^ab^	939 ^a^	5.26	0.025	922 ^b^	936 ^ab^	947 ^a^	4.33	0.028	3.59	0.001	0.02	0.862
CP	125	119	139	4	0.101	110 ^b^	132 ^ab^	152 ^a^	6.61	0.004	3.77	0.001	0.404	0.036
CT	136	138	119	3.77	0.052	116	121	109	2.31	0.122	2.87	0.007	<0.001	0.419
EE	18.6	18	21.3	0.927	0.341	24	21.6	27	1.35	0.306	0.991	0.112	0.009	0.856
NDF	365	369	361	4.78	0.834	352	380	372	6.48	0.211	3.92	0.303	0.73	0.381
ADF	199	208	201	3.07	0.485	186 ^b^	221 ^a^	207 ^a^	5.59	0.005	3.11	0.003	0.548	0.065
ADL	75.3	70.3	62.3	2.75	0.145	67.3 ^b^	83.0 ^a^	76.7 ^ab^	2.8	0.039	2.05	0.166	0.055	0.017
IVOMD	405 ^b^	443 ^a^	444 ^a^	7.19	0.008	413 ^b^	452 ^a^	457 ^a^	7.75	0.012	5.27	<0.001	0.095	0.936
ME	5.55 ^b^	6.62 ^a^	6.24 ^ab^	0.177	0.001	5.71 ^b^	6.46 ^a^	6.43 ^a^	0.147	0.027	0.112	0.001	0.637	0.511

DM, dry matter, OM, organic matter; CP, crude protein; CT, condensed tannins; EE, ether extract; NDF, neutral detergent fiber; ADF, acid detergent fiber; ADL, lignin; IVOMD, in vitro organic matter digestibility; ME, metabolizable energy; S, season; Y, year; SEM, standard error of the means. Within a row, values with different letters are significantly different (*p* < 0.05).

**Table 2:** Chemical composition (g/kg DM), IVOMD (g/kg), and ME (MJ/kg DM) of trees species (*n* = 4) and herbaceous browsed by goats in Southern Mediterranean forest rangeland of Northern Morocco during two contrasting years.

Item	2016 (Dry Year)					2017 (Wet Year)					SEM	*p*-Value (2016–2017)		
	Spring	Summer	Autumn	SEM	*p*-Value	Spring	Summer	Autumn	SEM	*p*-Value		S	Y	Y × S
*Olea europaea*														
DM	461 ^a^	437 ^b^	426 ^b^	5.57	0.003	444 ^a^	423 ^b^	414 ^b^	4.76	0.002	3.95	<0.001	<0.001	0.801
OM	954 ^a^	912 ^b^	907 ^b^	8.1	0.004	970 ^a^	920 ^b^	918 ^b^	8.67	0.001	5.94	<0.001	0.017	0.753
CP	76.3	79.3	82	2.86	0.773	66.7 ^b^	94.0 ^a^	89.3 ^a^	4.92	0.019	2.81	0.024	0.359	0.101
CT	4.20 ^a^	2.17 ^b^	3.20 ^ab^	0.32	0.004	3.87 ^a^	1.77 ^b^	2.63 ^ab^	0.327	0.002	0.228	<0.001	0.051	0.888
EE	94.0 ^b^	123 ^a^	79.6 ^c^	6.39	<0.001	99.0 ^b^	131 ^a^	85.0 ^c^	7.02	<0.001	4.67	<0.001	0.005	0.722
NDF	415 ^b^	449 ^a^	442 ^a^	5.72	0.005	404 ^b^	459 ^a^	450 ^a^	8.8	<0.001	5.1	<0.001	0.512	0.065
ADF	314 ^a^	258 ^b^	265 ^b^	9.74	0.008	302	279	276	5.29	0.067	5.44	<0.001	0.306	0.14
ADL	159	151	161	2.51	0.282	147 ^b^	164 ^a^	174 ^a^	4.41	0.008	2.53	0.011	0.178	0.012
IVOMD	499	517	517	4.28	0.105	505 ^b^	528 ^a^	530 ^a^	4.84	0.043	3.35	0.005	0.062	0.886
ME	6.85 ^c^	8.12 ^a^	7.59 ^b^	0.193	<0.001	6.96 ^b^	7.97 ^a^	7.75 ^a^	0.161	<0.001	0.122	<0.001	0.649	0.305
*Quercus canariensis*														
DM	564 ^c^	690 ^a^	634 ^b^	18.3	<0.001	548 ^c^	678 ^a^	620 ^b^	18.8	<.0001	12.8	<0.001	0.001	0.859
OM	939	961	963	4.91	0.054	950	967	974	4.38	0.054	3.41	0.003	0.058	0.871
CP	104 ^a^	63.7 ^c^	72.3 ^b^	6.23	<0.001	90.7 ^a^	77.3 ^b^	79.0 ^b^	2.22	0.001	3.22	<0.001	0.095	<0.001
CT	20	26.7	17.3	2.15	0.194	14	16.3	12.7	1.09	0.438	1.45	0.081	0.008	0.554
EE	18.2 ^b^	24.6 ^a^	24.0 ^a^	1.18	0.015	21.7	27.3	28	1.3	0.075	0.947	0.002	0.014	0.897
NDF	488 ^c^	550 ^a^	525 ^b^	9.25	<0.001	480 ^c^	560 ^a^	535 ^b^	11.9	<0.001	7.33	<0.001	0.157	0.036
ADF	322 ^c^	372 ^b^	394 ^a^	10.7	<0.001	317 ^c^	382 ^b^	404 ^a^	13	<0.001	8.19	<0.001	0.01	0.003
ADL	114 ^c^	157 ^b^	176 ^a^	9.44	<0.001	103 ^c^	168 ^b^	189 ^a^	13.1	<0.001	7.85	<0.001	0.255	0.025
IVOMD	602 ^a^	406 ^c^	446 ^b^	30	<0.001	607 ^a^	414 ^c^	454 ^b^	29.3	<0.001	20.4	<0.001	0.007	0.694
ME	8.69 ^a^	6.00 ^b^	6.30 ^b^	0.427	<0.001	8.82 ^a^	5.81 ^c^	6.42 ^b^	0.462	<0.001	0.305	<0.001	0.775	0.159
*Quercus ilex*														
DM	571 ^b^	612 ^a^	601 ^a^	6.25	<0.001	551 ^b^	596 ^a^	588 ^a^	7.14	<0.001	5.02	<0.001	<0.001	0.509
OM	943	953	957	3.34	0.251	955	960	968	3.36	0.322	2.59	0.086	0.048	0.926
CP	114 ^a^	70.3 ^b^	71.3 ^b^	7.6	0.001	99.7 ^a^	83.0 ^ab^	78.0 ^b^	4.03	0.039	4.18	<0.001	0.687	0.029
CT	26.3 ^b^	60.0 ^a^	55.0 ^a^	5.31	<0.001	22.7 ^b^	48.3 ^a^	45.7 ^a^	4.17	<0.001	3.42	<0.001	<0.001	0.097
EE	17.7	19.1	19	0.83	0.803	19.6	22.6	23.6	1.1	0.35	0.786	0.302	0.034	0.736
NDF	568 ^a^	534 ^b^	506 ^b^	9.82	0.004	553 ^a^	539 ^ab^	512 ^b^	7.58	0.045	6.02	<0.001	0.889	0.399
ADF	352	322	333	5.77	0.071	342	334	345	3.91	0.559	3.44	0.063	0.416	0.256
ADL	170	163	171	3.18	0.582	162 ^b^	175 ^ab^	192 ^a^	5.16	0.024	3.13	0.039	0.083	0.071
IVOMD	508 ^a^	410 ^c^	459 ^b^	14.4	<0.001	513 ^a^	424 ^c^	468 ^b^	13.1	<0.001	9.49	<0.001	0.049	0.673
ME	7.16 ^a^	5.99 ^c^	6.59 ^b^	0.172	<0.001	7.38 ^a^	5.87 ^c^	6.70 ^b^	0.223	<0.001	0.137	<0.001	0.317	0.146
*Quercus suber*														
DM	587 ^b^	650 ^a^	604 ^b^	9.7	<0.001	573 ^b^	639 ^a^	589 ^b^	10.2	<0.001	7.03	<0.001	0.004	0.932
OM	968 ^a^	957 ^ab^	947 ^b^	3.8	0.04	978	969	963	3.5	0.212	2.94	0.01	0.009	0.829
CP	85	78.3	88.3	2.36	0.229	75.3	91	95	3.83	0.057	2.22	0.056	0.374	0.056
CT	119	132	124	2.93	0.196	110	118	116	2.7	0.48	2.29	0.11	0.02	0.796
EE	25	26	27.3	1.12	0.752	28.6	29.3	30.3	1.09	0.86	0.861	0.653	0.081	0.988
NDF	579 ^a^	502 ^b^	485 ^b^	14.7	<0.001	565 ^a^	511 ^b^	490 ^b^	11.5	0.004	9.07	<0.001	0.982	0.164
ADF	377 ^a^	348 ^b^	311 ^c^	10.1	0.001	367 ^a^	367 ^a^	321 ^b^	8.22	0.003	6.36	<0.001	0.223	0.097
ADL	168 ^a^	134 ^b^	133 ^b^	6.12	0.002	161	146	149	2.98	0.069	3.41	<0.001	0.061	0.036
IVOMD	543 ^a^	406 ^c^	506 ^b^	20.4	<0.001	550 ^a^	421 ^c^	513 ^b^	19.2	<0.001	13.6	<0.001	0.003	0.412
ME	8.05 ^a^	6.00 ^c^	7.34 ^b^	0.296	<0.001	8.19 ^a^	5.84 ^c^	7.51 ^b^	0.353	<0.001	0.223	<0.001	0.605	0.085
Herbaceous														
DM	463 ^c^	631 ^a^	516 ^b^	25	<0.001	446 ^c^	616 ^a^	495 ^b^	25.6	<0.001	17.5	<0.001	0.019	0.923
OM	916 ^a^	870 ^b^	855 ^b^	9.8	0.002	931 ^a^	883 ^b^	872 ^b^	9.5	0.001	6.87	<0.001	0.013	0.958
CP	156 ^a^	78.3 ^b^	65.7 ^b^	14.2	<0.001	142 ^a^	91.0 ^b^	76.7 ^b^	10.1	<0.001	8.45	<0.001	0.291	0.006
CT	2.42 ^b^	4.17 ^a^	2.97 ^b^	0.323	0.048	2.2	3.53	2.43	0.287	0.116	0.217	0.006	0.176	0.865
EE	19.9	22	23.3	0.823	0.262	22.6	26.3	26.3	1.12	0.349	0.789	0.11	0.027	0.872
NDF	517	568	497	15.4	0.147	508	580	507	16.2	0.087	10.8	0.014	0.827	0.865
ADF	339 ^b^	363 ^a^	269 ^c^	14.7	0.001	326 ^b^	379 ^a^	283 ^c^	14.6	0.001	10.1	<0.001	0.426	0.216
ADL	71.4	75.7	64.3	2.76	0.265	67.0 ^b^	89.0 ^a^	79.0 ^ab^	3.95	0.043	2.53	0.031	0.056	0.106
IVOMD	804 ^a^	651 ^c^	705 ^b^	22.7	<0.001	807 ^a^	658 ^c^	719 ^b^	22	<0.001	15.3	<0.001	0.223	0.771
ME	12.0 ^a^	9.25 ^c^	10.7 ^b^	0.405	<0.001	12.1 ^a^	9.57 ^c^	10.8 ^b^	0.364	<0.001	0.265	<0.001	0.097	0.334

DM, dry matter, OM, organic matter; CP, crude protein; CT, condensed tannins; EE, ether extract; NDF, neutral detergent fiber; ADF, acid detergent fiber; ADL, lignin; IVOMD, in vitro organic matter digestibility; ME, metabolizable energy; S, season; Y, year; SEM, standard error of the means. Within a row, values with different letters are significantly different (*p* < 0.05).

## Data Availability

The data presented in this study are available from the corresponding author on request.
